# Supplementary material for: Demonstration of a quantum C-NOT gate in a time-multiplexed fully reconfigurable photonic processor
Source: Nat Commun. 2026 Jun 30;17:5683. doi: 10.1038/s41467-026-74861-9 (PMC13320172; doi:10.1038/s41467-026-74861-9)
Supplement: Supplementary file 1 — Supplementary Information [file 41467_2026_74861_MOESM1_ESM.pdf]

Supplementary Notes to: Demonstration of a quantum C-NOT Gate in a Time-Multiplexed fully reconfigurable photonic processor

Federico Pegoraro, Philip Held, Jonas Lammers, Benjamin Brecht, and Christine Silberhorn
Paderborn University, Integrated Quantum Optics,
Institute of Photonic Quantum Systems (PhoQS) Warburger Str. 100, 33098, Paderborn, Germany

Supplementary Note 1. SPDC SOURCE

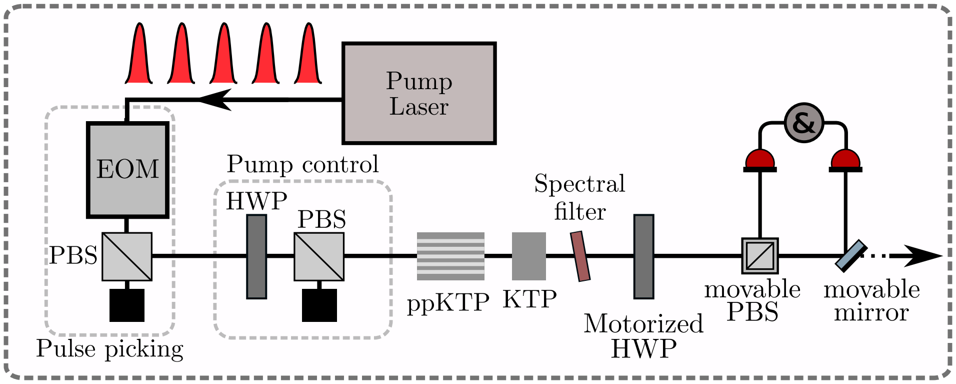

Supplementary Fig. 1. Photon pairs are produced in a periodically poled potassium titanyl phosphate (ppKTP) waveguide and spectrally filtered. The second KTP crystal compensates the walk-off between the signal and the idler photon in the type II process. The EOM in the pump path picks the pump pulses and controls the repetition rate of the experiment, while input power and polarization are controlled by an HWP and a PBS.
A motorized HWP combined with a movable PBS and mirror can be used to perform polarization HOM interference. Abbreviations: electro-optic modulator (EOM), polarization beam-splitter (PBS), half-waveplate (HWP).

The two photons are produced using a type-II parametric down-conversion (PDC) source based on a periodically poled potassium-tytanil phosphate (ppKTP) waveguide. Using this process we can produce a pair of signal-idler photons with orthogonal polarizations and high spectral and spatial purity. Supplementary Fig. 1 shows a scheme of the experimental setup employed in order to generate the photon pairs. We pump the process with a pulsed laser with a central wavelength of 772.5 nm, a bandwidth of approx. 0.3 nm, and a repetition rate of 76.4 MHz. In order to reduce the repetition rate we perform pulse picking, to this aim we use a fast electro-optical modulator (EOM) acting on the pump polarization. The incoming laser pulses enter this EOM in a vertically polarized state and are sent to a beam dump positioned after a polarization beam-splitter (PBS) unless a voltage is applied to the Pockels cell inside the modulator. Synchronizing the application of voltage to the EOM to the arrival time of the laser pulses we can switch polarization of each $n-th$ pulse from H to V and direct it to the rest of the setup, thus reducing the repetition rate by a factor equal to n. After picking a pulse we adjust its power by means of a power control consisting of a half-wave plate and a PBS. At this point the pump laser is in-coupled to a 25 mm long ppKTP waveguide. As stated in the main text, pump power is set to a level resulting in a mean photon number of 0.01 in order to limit events where more than two photons are generated. The partner photons are generated at a wavelength of approx. 1550 nm and a temporal duration of approx. 3.2 ps, in order to improve spectral purity and indistinguishability we apply a spectral filter with 1.8 nm FWHM bandwidth which is angle tuned to a central wavelength of 1545.22 nm. In order to ensure the temporal overlap of the two photons, an unpoled, 10.7 mm long KTP crystal is positioned in between waveguide and spectral filter. Setting the fast axis of this compensation crystal to form an angle of 90° with respect to the one of the waveguide we compensate for the temporal walk-off between signal and idler induced by the waveguide birefringence. In order to find the proper length for the compensation crystal we performed polarization HOM interference with KTP crystal of different lengths and recorded the visibilities, which are plotted in Supplementary Fig. 2a) as a function of the KTP length. Fitting a Gaussian to the visibilities we obtain that a crystal 10.7 mm long is expected to realise an optimal temporal overlap. Therefore, we acquired a crystal with this length and used it to perform again polarization HOM. To this aim, we make use of the motorized HWP shown in Supplementary Fig. 1 in combination with a movable PBS and mirror, which are used to separate the two polarizations and direct them to two SNSPDs.

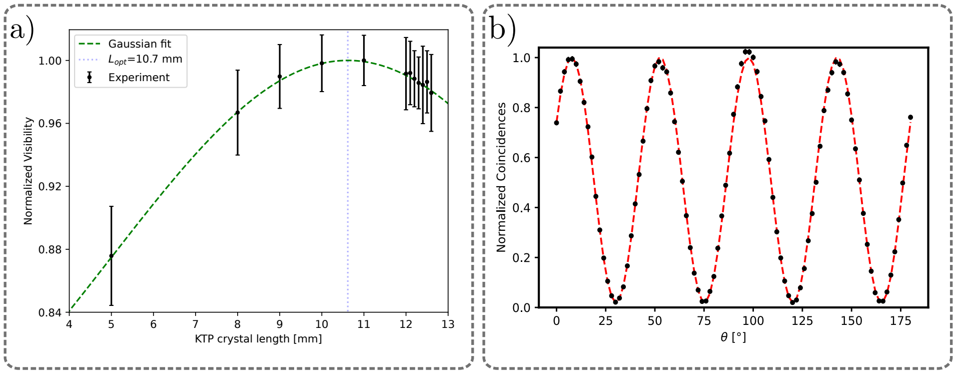

Supplementary Fig. 2. a) Visibility of the Hong-Ou-Mandel interference as a function of the KTP compensation crystal length, fitting the obtained nomalized visibilites to a Gaussian function we expect an optimal visibility for a crystal length of 10.7 mm. The error bars are calculated fitting a $\cos^2$ function to the individual coincidence scans. b) Polarization Hong-Ou-Mandel interference of the signal and idler photon generated in the type II PDC source driven at a mean photon number of $\langle n\rangle = 0.01$. From a $\cos^2$ we derive a raw visibility of $(98 \pm 1)\%$. Error bars are calculated from the Poissonian error associated to the raw coincidences.

After passing through the HWP signal and idler enter the PBS from a singe port and the interference trace is obtained by recording coincidences between the two PBS outputs at different angles of the HWP. In Supplementary Fig. 2b) we show the polarization HOM scan performed with the aforementioned compensation crystal, for which we obtain fringes whose visibility is extracted by fitting a $\cos^2$ function to the normalized coincidences. In this way we find the HOM visibility of the generated photons to be $(98 \pm 1)\%$.

Supplementary Note 2. GATE FIDELITY BOUNDS

In the following we expand on effects of experimental imperfections outside time multiplexed C-NOT circuit limiting the maximum fidelity for the reconstructed gate logic table. As stated in the main text, we employ a Type-II SPDC process pumped with a single pulse to generate our control-target (C-T) pair consisting of a two mode squeezed state. Using the scheme discussed in the Results section, we can route the signal and idler photons to the desired C-T input combination. As a first consideration, we must take into account that the spectral properties of both signal and idler fields are not completely identical. Hence, the two will not be perfectly indistiguishable. We quantified the signal-idler degree of indistinguishbility using HOM interference obtaining a visibility $V = (98 \pm 1)\%$. We start by considering the expression of the two-mode squeezed state in terms of photon number states, which can be written as:

$$|\Psi\rangle = \sqrt{1-|\lambda|^2}\sum_{n=0}^{\infty}\lambda^n\left(\sqrt{V}\hat{a}_c^\dagger + \sqrt{1-V}\hat{b}_c^\dagger\right)^n\left(\hat{a}_t^\dagger\right)^n|\varnothing\rangle, \tag{1}$$

where $|\varnothing\rangle$ is the vacuum state, $\hat{a}^\dagger$ and $\hat{b}^\dagger$ are the creation operators associated to indistinguishable and distinguishable photons, respectively. V is the degree of indistinguishability between signal and idler and λ regulates the relative weight between contributions corresponding to different number of photons present in the signal-idler pair. The absolute value of λ can be tuned by setting the power of the pump field. Since we want to use a photon pair to encode the input state of the gate, we use this dependence to limit the probability of producing more than two photons per generation. By imposing a mean photon number of 0.01 photons per generation we expect the probability of a two photon event to be $P(2) \approx 98\%$ of all successful generations instances, while the probability for a four photons event will be $P(4) \approx 2\%$ and higher contributions can be neglected.
Considering the transmission, round-trip and detection efficiencies of our setup, the probability that a two-photon state is detected after the 6 round-trips required to implement the C-NOT gate is $\approx 0.33\%$. Of all the detected two photon events, we expect a fraction V to come from indistinguishable particles. If we assume a perfect C-NOT gate, all the indistinguishable events will give correct contributions in the output truth table, while the distinguishable can result incorrect outcomes depending on the C input state. The C-NOT circuit has a success probability of $\frac{1}{9}$, thus two indistinguishable photons have a probability $P(2)P_D(2)V\frac{1}{9} \approx 0.036\%$ of being measured in the correct output mode combination. On the other hand, distinguishable contributions have a probability of $\approx 0.00054\%$ to give valid pattern to the C-NOT truth table when C is initiated in the $|0\rangle$ state. Conversely, if the C qubit starts in the $|1\rangle$

state, then pairs of distinguishable photons can contribute to both the desired output pattern with a probability of approx. 0.00054%, as well as giving wrong output patterns with a probability of approx. 0.0011%.

In addition to errors coming from non-perfect indistinguishabilty in the two-photon case, we simulate the effect of four-photon states coming from the source and propagating in the interferometer. We include this contribution in our analysis because our detectors cannot resolve the number of photons that triggered a detection instance, thus there is the possibility of reading out a C-T coincidence event coming from a four-photon input without being able to distinguish it from a true two-photon contribution. In this case we restrict the analysis only to the indistinguishable case as the probability of a contribution from a four-photon event with distinguishable photons is negligible. Finally we take into account that, due to imperfections in the detection part of our setup, we have a polarization resolution error resulting in a 1% probability for a qubit in the $|1\rangle$ state to be wrongly detected as a $|0\rangle$.

Considering all the above factors, for each input C-T combination we can calculate the probabilities for correct or wrong outcomes both for two- and four-photons generation events in the distinguishable (dist) and indistinguishable (indis), which we list in Supplementary Tab. 1.

	$(c=0,t=0)$	$(c=0,t=1)$	$(c=1,t=0)$	$(c=1,t=1)$
Correct $P(n=2,\text{indis})$	0.036%	0.036%	0.036%	0.036%
Error $P(n=2,\text{indis})$	0%	0.00036%	0.00072%	0%
Correct $P(n=2,\text{dis})$	0.0017%	0.0010%	0.00061%	0.0017%
Error $P(n=2,\text{dis})$	0%	0.000010%	0.0012%	0.0011%
Correct $P(n=4,\text{indis})$	0.0019%	0.0019%	0.0015%	0.0015%
Error $P(n=4,\text{indis})$	0.00094%	0.00095%	0.0011%	0.0010%
$P_{Success}$	0.039%	0.038%	0.037%	0.039%
P_{Error}	0.00094%	0.0013%	0.0029%	0.0021%
P_{C-NOT}	98%	97%	93%	95%

Supplementary Table 1. Probabilities for different input patterns depending on photon number and indistinguishability. For all cases we consider the probability for a given contribution of the PDC to give a correct outcome or an error. The overall probability for success and error a are obtained by summing the individual contributions. $P_{\text{C-NOT}}$ is the probability that the input state is transformed into the expected logical output, assuming an ideal C-NOT gate.

The overall probability for correct or wrong outcomes is then obtained by summing the individual contributions, while the probability of observing the expected C-NOT outcome given an imperfect input state is given by

$$\mathrm{P}_{\text{C-NOT}} = \frac{P(\text{Success})}{P(\text{Success}) + P(\text{Error})}. \tag{2}$$

As it emerges from this analysis, the two c_0 inputs feature working probabilities of 97% and 98%, whereas in the c_1 cases the working probabilities are bound to 93% and 95%, the reduced probability for latter case has to be ascribed to the interplay between indistinguishability and higher photon number contributions. By averaging on the estimated working probabilities we can give an upper bound for the fidelity of the C-NOT operation for which we obtain a value of $F_{\max} = 95.5\%$.

Supplementary Note 3. TWO-QUBIT TOMOGRAPHY SETUP

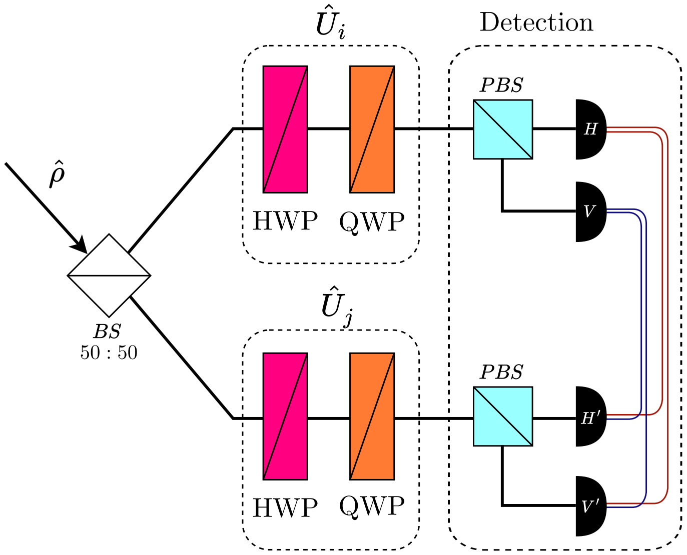

Supplementary Fig. 3. Sketch of the tomography setup. The 50:50 BS splits photons probabilistically and sends them to two units consisting of an half- and quarter-waveplate that rotate the state into the base that diagonalizes $\hat{\sigma}_i \otimes \hat{\sigma}_j$. After each unit a PBS separates H and V contributions and by correlating the outcomes of first and second unit it is possible to reconstruct $\langle \hat{\sigma}_i \otimes \hat{\sigma}_j \rangle$. Abbreviations: beam-splitter (BS), half-waveplate (HWP), quarter-waveplate (QWP).

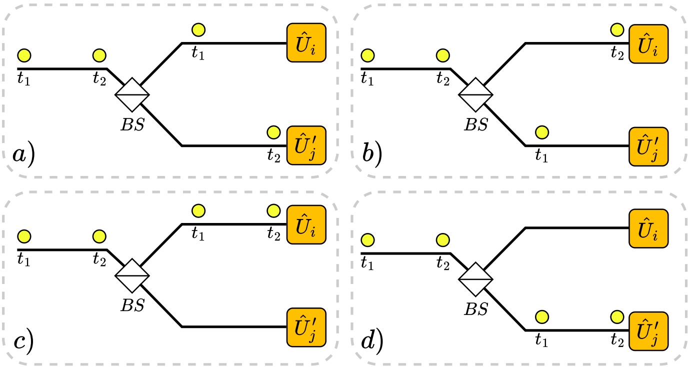

Supplementary Fig. 4. Starting with two photons in two time-bins t_1 and t_2, the four outcomes a)-d) after the BS are possible. The two time-bins are either separated (cases a) and b)) or sent to the same detection unit (cases c) and d)). Depending on the specific outcome the state is projected on the eigenbasis of $\hat{\sigma}_i \otimes \hat{\sigma}_j$, $\hat{\sigma}_j \otimes \hat{\sigma}_i$, $\hat{\sigma}_i \otimes \hat{\sigma}_i$ and $\hat{\sigma}_j \otimes \hat{\sigma}_j$. Abbreviations: beam-splitter (BS).

Supplementary Fig. 3 shows a sketch of the tomography setup. Photons coming from the TM setup travel to a 50:50 BS, after which two combinations of a half- and quarter-waveplate (HWP and QWP) installed into motorized rotation mounts implement projections on the Pauli bases according to the angle settings listed in Supplementary Tab. 2. Each combination implements a tomographic unitary $\hat{U}_i$, with $i = x, y, z$, which rotates the local polarization

	HWP	QWP
$\hat{\sigma}_x$	22.5°	0°
$\hat{\sigma}_y$	45°	-45°
$\hat{\sigma}_z$	0°	0°

Supplementary Table 2. Angle settings for the two tomographic units

from the basis that diagonalizes $\hat{\sigma}_i$ to the H-V one. This allows to implement the projective measurement in the selected basis, using a PBS to separate H and V polarized light and direct it to two SNSPDs.
The expectation values $\langle\hat{\sigma}_i \otimes \hat{\sigma}_j\rangle$ are obtained using many copies of $\hat{\rho}$ and measuring coincidences between detector pairs: HH', HV', VH' and VV', whereby:

$$\langle\hat{\sigma}_i \otimes \hat{\sigma}_j\rangle = \frac{C^{(i,j)}_{HH'} - C^{(i,j)}_{HV'} - C^{(i,j)}_{VH'} + C^{(i,j)}_{VV'}}{C^{(i,j)}_{HH'} + C^{(i,j)}_{HV'} + C^{(i,j)}_{VH'} + C^{(i,j)}_{VV'}}, \tag{3}$$

where the superscripts indicate the combination of unitaries applied to the photons. A complete reconstruction of $\hat{\rho}$ requires measuring expectation values of the form: $\langle\mathbb{I} \otimes \hat{\sigma}_j\rangle$, $\langle\hat{\sigma}_i \otimes \mathbb{I}\rangle$ and $\langle\mathbb{I} \otimes \mathbb{I}\rangle$, where $\mathbb{I}$ is the 2×2 identity. While the latter may be obtained as a normalization form all the others, the former two can be still obtained as the $\langle\hat{\sigma}_i \otimes \hat{\sigma}_j\rangle$ combining coincidences as follows:

$$\langle\hat{\sigma}_i \otimes \mathbb{I}\rangle = \frac{C^{(i,i)}_{HH'} + C^{(i,i)}_{HV'} - C^{(i,i)}_{VH'} - C^{(i,i)}_{VV'}}{C^{(i,i)}_{HH'} + C^{(i,i)}_{HV'} + C^{(i,i)}_{VH'} + C^{(i,i)}_{VV'}}, \tag{4}$$

$$\langle\mathbb{I} \otimes \hat{\sigma}_j\rangle = \frac{C^{(j,j)}_{HH'} - C^{(j,j)}_{HV'} + C^{(j,j)}_{VH'} - C^{(j,j)}_{VV'}}{C^{(j,j)}_{HH'} + C^{(j,j)}_{HV'} + C^{(j,j)}_{VH'} + C^{(j,j)}_{VV'}}. \tag{5}$$

The density operator $\hat{\rho}$ is obtained considering that a generic two-qubit state may be written as $\hat{\rho} = \sum_{ij} s_{i,j}\hat{\sigma}_i \otimes \hat{\sigma}_j$, with $i = 0, x, y, z$ and $\hat{\sigma}_0 = \mathbb{I}$.
To be able to reconstruct $\hat{\rho}$ it is necessary to perform a sufficient amount of measurements, *i.e.* : set enough combinations of $\hat{U}_i$ and $\hat{U}_j$. Considering a case where the two photons encoding the two qubit state $\hat{\rho}$ occupy two time-bins t_1 and t_2, upon reaching the 50:50 BS, the four outcomes shown in Supplementary Fig. 4 are possible. Accordingly, the two photons are either separated (cases a) and b)) or directed to the same tomographic unit (cases c) and d)). In cases a) and b) the state is projected on the eigenbasis of $\hat{\sigma}_i \otimes \hat{\sigma}_j$ and $\hat{\sigma}_j \otimes \hat{\sigma}_i$, while in the two remaining cases $\hat{\rho}$ is projected on the eigenbasis of $\hat{\sigma}_i \otimes \hat{\sigma}_i$ and $\hat{\sigma}_j \otimes \hat{\sigma}_j$. Therefore, if $t_1 \neq t_2$, the three settings: $(\hat{U}_z, \hat{U}_x)$, $(\hat{U}_z, \hat{U}_y)$ and $(\hat{U}_x, \hat{U}_y)$ are sufficient to reconstruct the state postselecting on events matching the four possible outcomes.
It is possible to reconstruct $\hat{\rho}$ even when $t_1 = t_2$, however, in this case all outcomes where the two photons are not separated become unusable as our detectors do not have photon number resolution. For this reason to reconstruct the state the settings: $(\hat{U}_z, \hat{U}_z)$, $(\hat{U}_x, \hat{U}_x)$ and $(\hat{U}_y, \hat{U}_y)$ must be added to the ones seen for the previous case. This allows to reconstruct the state by postselecting only on outcomes where photons were separated by the 50:50 BS.

Supplementary Note 4. RECONSTRUCTED BELL STATES

We compare the reconstructed Bell states generated using the TM setup, which are shown in Supplementary Fig. 5, to the theoretically expected ones using the quantum state fidelity

$$\mathcal{F}_\psi = \langle\psi \mid \hat{\rho}_e \mid \psi\rangle, \tag{6}$$

where $|\psi\rangle$ here represents a pure state of two-qubits and $\hat{\rho}_e$ the experimentally reconstructed density operator. In our tomography setup, we measure the correlations among Pauli operators

$$s_{i,j} = \langle\hat{\sigma}_i \otimes \hat{\sigma}_j\rangle, \tag{7}$$

with $i = 0, x, y, z$ and $\hat{\sigma_0}$ denoting the 4×4 identity.
The experimentally reconstructed density operator can be written as

$$\hat{\rho}_e = \sum_{i,j} \frac{s_{i,j}}{4}\,\hat{\sigma}_i \otimes \hat{\sigma}_j. \tag{8}$$

Accordingly, the fidelity becomes

$$\mathcal{F}_\psi = \sum_{i,j} \frac{s_{i,j}}{4}\,\langle\psi \mid \hat{\sigma}_i \otimes \hat{\sigma}_j \mid \psi\rangle. \tag{9}$$

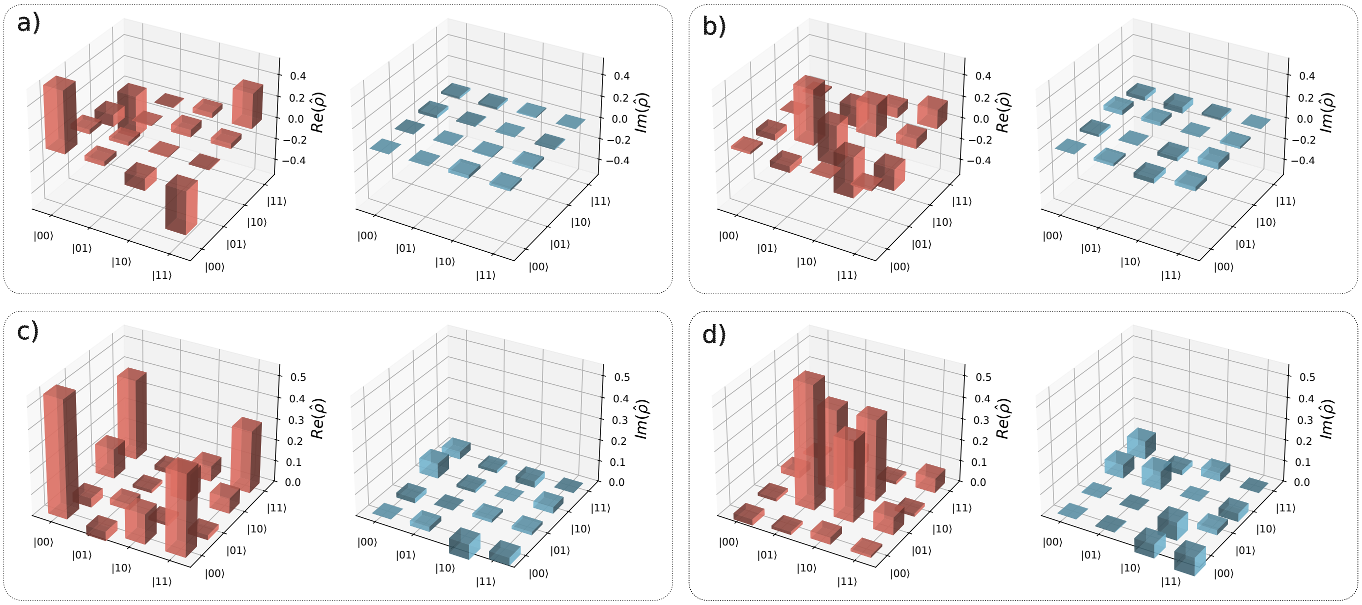

Supplementary Fig. 5. Reconstructed real and imaginary part of the Bell states obtained combining the TM C-NOT and an Hadamard gate. The four panels show the states: a): $|\phi^-\rangle$ b): $|\psi^-\rangle$ c): $|\phi^+\rangle$ d): $|\psi^+\rangle$, with quantum state fidelities:$\mathcal{F}_{\Psi^-} = (78.1 \pm 2.8)\%$, $\mathcal{F}_{\Psi^+} = (85.6 \pm 3.0)\%$, $\mathcal{F}_{\Phi^-} = (85.0 \pm 2.4)\%$ and $\mathcal{F}_{\Phi^+} = (80.3 \pm 3.0)\%$

Given the uncertainties $\Delta s_{i,j}$ associated with the measured correlations, which can be obtained propagating the Poissonian error of the coincidence counts employed to calculate the correlations (see Supplementary Eqs. 3-5), the uncertainty in $\mathcal{F}_\psi$ can be obtained via standard Gaussian error propagation as $\Delta\mathcal{F}_\psi^2 = \sum_{i,j}(\frac{\partial \mathcal{F}_\psi}{\partial s_{i,j}})^2 \Delta s_{i,j}^2$ and thus,

$$\Delta\mathcal{F}_\psi = \sqrt{\sum_{i,j} \frac{\langle \psi \mid \sigma_i \otimes \sigma_j \mid \psi \rangle^2}{16} \Delta s_{i,j}^2}. \tag{10}$$

Using Supplementary Eqs. (6) and (10), we find the quantum state fidelities and respective errors for the experimentally generated Bell states:

$$\mathcal{F}_{\psi^+} = (85.6 \pm 3.0)\%,$$

$$\mathcal{F}_{\psi^-} = (78.1 \pm 2.8)\%,$$

$$\mathcal{F}_{\phi^+} = (80.3 \pm 3.0)\%,$$

$$\mathcal{F}_{\phi^-} = (85.0 \pm 2.4)\%.$$

Supplementary Note 5. INFLUENCE OF LOSSES IN THE TM SETUP AND ACHIEVABLE TWO-PHOTON RATES

Although the TM architecture provides a platform capable of implementing large interferometers in a resource-efficient manner, particularly in terms of the number of required active elements, it is especially sensitive to losses in

the two loops. In particular, the probability that a single photon from the source is detected after n round-trips in TM setup is given by

$$p_n = \eta_0 \cdot \eta_{\text{loop}}^{n-1}, \tag{11}$$

where η_0 is the transmission efficiency from generation to detection considering a single pass through the TM setup, and η_{loop} is the average loop efficiency. In the setup presented in the main text we have $\eta_0 = 0.21$ and $\eta_{\text{loop}} = 0.81$.

For a two-photon evolution in which the source is set to a mean photon-pair number $\langle n \rangle$ at a repetition rate ν_{rep}, the expected number of two-photon events after n round-trips, integrated over a time τ_{int}, is given by

$$C_n = \langle n \rangle \cdot \nu_{\text{rep}} \cdot \tau_{\text{int}} \cdot p_n^2. \tag{12}$$

In our case, $\langle n \rangle = 0.01$ and $\nu_{\text{rep}} = 21$ kHz. Considering an integration time of one hour, the setup produces, after n round-trips, the number of two-photon events listed in Supplementary Tab. 3. From this table, we observe that for the loop efficiency of the current setup, and without accounting for post-selection, approximately 4000 coincidence events are expected after six round-trips under the aforementioned experimental conditions.

**Round-trip**	$\eta_{\text{loop}} = 81\%$	$\eta_{\text{loop}} = 85\%$	$\eta_{\text{loop}} = 89\%$
**1**	33340	33340	36479
**2**	21874	24088	28838
**3**	14352	17403	22797
**4**	9416	12574	18022
**5**	6178	9085	14247
**6**	4053	6564	11263

Supplementary Table 3. Achievable two-photon rates in the TM setup for different loop efficiencies of 81%, 85% and 89% considering a repetition rate of 21 kHz and an integration time of 1 hour and a mean photon number of 0.01 for the source. The n-th row of the table corresponds to the output of the n-th round-trip.

Since, as shown in Supplementary Eq. 11, the loop efficiency enters the loss budget exponentially, achieving higher values of η_{loop} is expected to dramatically improve the achievable two-photon rates. A first factor limiting the loop efficiency is the quality of the mode matching between the fiber collimators used to couple light into the delay fibers. Because of the physical size of the EOMs, the distance between the collimators cannot be reduced below 80 cm. The fiber collimators have a focal length of 8 mm; this value is chosen to ensure a sufficiently small beam waist, which is required both for the reliable operation of the modulators and to render it compatible with the clear aperture of the Pockels cells. Considering that the single-mode fibers used to implement the loops have a mode-field diameter of 10 μm, and adopting standard Gaussian beam propagation, we find that upon re-entering the fiber the overlap between beam and fiber mode is 91.5%. By adopting newer free space EOMs with a reduced footprint, which are already commercially available, it would be possible to reduce the distance between the collimators to 55 cm. This would increase the mode overlap to 95.9%, allowing a loop efficiency of approximately 85%. Keeping all other experimental parameters unchanged, the setup would then be expected to produce the two-photon rates listed in the second column of Supplementary Tab. 3. Please note that, in this case, η_0 remains unchanged and no light is coupled back into the fibers after the first round trip. Consequently, from Supplementary Eqs. 11 and 12, the expected two-fold event rate would be the same as in the previous case. However, the effect of improved mode matching becomes apparent when considering higher round-trip numbers. In particular, this modification alone would provide an improvement of about 60% in the rates after six round-trips and would reduce the required integration time by a factor of 1.6.
In addition, another significant contribution to the system losses arises from attenuation in the fibers. Taking into account a typical attenuation of 0.2 dB/km for single-mode fibers at 1550 nm, the transmission through the long fiber in our setup (1120 m) cannot exceed approximately 95%. As pointed out in the main text, the adoption of faster EOMs with bandwidths up to 100 MHz, which may become available in the foreseeable future, would allow the fiber lengths to be reduced by approximately one order of magnitude. This would increase the upper bound for the fiber transmission efficiency to about 99.3%. By rescaling both η_0 and η_{loop} accordingly, it would then be possible to achieve the two-photon rates listed in the third column of Supplementary Tab. 3. In this scenario, after six round-trips we would expect count rates approximately three times higher than those achieved with the current setup. Combined with the fact that the system could be operated at a repetition rate ten times larger because of the shorter delay lines, the overall data rate could be increased by a factor of about 30, significantly reducing the integration time required to collect the same amount of data.
