# Supplementary material for: Demonstration of a quantum C-NOT gate in a time-multiplexed fully reconfigurable photonic processor
Source: Nat Commun. 2026 Jun 30;17:5683. doi: 10.1038/s41467-026-74861-9 (PMC13320172; doi:10.1038/s41467-026-74861-9)
Supplement: Supplementary file 2 — Transparent Peer Review file [file 41467_2026_74861_MOESM2_ESM.pdf]

nature portfolio

Peer Review File

Demonstration of a quantum C-NOT Gate in a Time-Multiplexed fully reconfigurable photonic processor

Corresponding Author: Dr Federico Pegoraro

This file contains all reviewer reports in order by version, followed by all author rebuttals in order by version.

Version 0:

Reviewer comments:

Reviewer #1

(Remarks to the Author)
For Authors,
I think this is an interesting research topic, namely demonstrating the C-NOT gate using a time-multiplexing (TM) system, even though the same research group has already used this system in quantum walk experiments (only 1/2 beam splitter). The experimental arguments of the two studies were different, so I expected to find a different detailed discussion than in the previous study. However, the manuscript is not clear to follow. I suggest that the author use figures and small subsections to explain 1. how their different beam splitters work with EOM, 2. how to operate the C-NOT gate through the TM system, and 3. how to generate entanglement with the C-NOT gate, and make their structure strongly connected to each part.

This is an experimental study, but it didn't clearly show its experimental details, for example, how to generate the SPDC photon source, laser information, how to operate EOMs in each round-trip, the length of the fiber,..., which makes it difficult to judge.

TM in Fig. 2 is very nice, but it didn't clearly explain how to operate and connect the experimental method with Fig. 1. If the authors can describe each specific part, such as Ref. [21], PRL 104, 050502 (2010), [23], NJP, 18, 063017 (2016), and [26], PRL 111, 150501 (2013), and the related output time-bin as shown in Fig. 1 (b). That would be better for the reader to easily understand how it worked.

The operation for each EOM is important, depending on every time-bin photon, so I recommend that authors use additional figures and paragraphs to clearly describe it, or use one specific input to demonstrate how it works. For the time-bin logic gates, they need to use a high-speed optical switch for every time-bin state, but the loss is also limited to their application. What is its operation limit and loss of EOM?

The authors discussed the errors of input control and target states, 0 and 1. For the TM, authors can check the result for all 6 round-trip distributions for C-NOT gate operation and entanglement generation. Another possible error may arise from the light propagation through long optical fibers and EOMs after 6 round-trip. How are these possible to affect the demonstrated results?

Do the 98% HOM interference visibility and C-NOT (entanglement generation) experiments use the same parameters, mean photon number of ~0.01? The SPDC photon indistinguishability is 98% (HOM), but the gate operation fidelity is 93.8% (Fig. 3) and is limited to 95% by the analysis. What is the main expected difference from the perfect gate? From Fig. 3 and Table I results, it does not seem consistent. For c=0, Fig.3 both are higher than Table I; for c=1, both are lower than Table I. c=1, t=0 case has the max difference. What is the error bar of the operation probabilities of probability in Fig. 3?

The best way to fully reconstruct the gate is quantum process tomography, but it needs lots of measurements. If the author performed these measurements, Phys. Rev. Lett. 94, 160504 (2005)., and Phys. Rev. Lett. 95, 210506 (2005)., it can give the bounds of the logic gate easily.

If authors can provide the data of Fig. 4 (a), it will be easy to confirm the average fidelity of 92.6%.

From the discussion about the C-NOT gate operation, it showed that the logic gate fidelity is very close to perfect at 95%,

and the SPDC photons are also very close to indistinguishability. What is the main reason that the state fidelity of generated Bell states became lower, and the error bar is also large in Fig. 4? If the C-NOT gate works well, is the quality of the specific input state good (input preparation)?

The authors talk about the distinguishability and indistinguishability of SPDC photons. I suggest the authors can also discuss more about the C-NOT gate itself to the possible error in the TM system.

I also recommend that authors survey more references about the photonic logic gates in different systems. And also introducing more about the advantages of using the TM system for PQC and other possible applications.

If possible, please reconsider the structure of the manuscript; the most recently submitted version is difficult to follow. For example, divide the results section into several subsections and ensure a natural transition between them, including appropriate figures and captions.

The material in the supplemental information is strongly related to the main text. I think it is better to move it to the method section or the main text.

Reviewer #2

(Remarks to the Author)
Review of "Demonstration of a quantum C-NOT Gate in a Time-Multiplexed fully reconfigurable photonic processor" by Federico Pegoraro et al.

The above manuscript describes the demonstration of a quantum controlled NOT gate. Here the target and control qubit are encoded in the in the time bin degree of freedom, and the entire circuit is constructed in a multi-pass configuration with electro-optic modulators (EOMs) providing programable beam splitter ratios and phases.

I find the approach to be elegant, and the gate fidelity compares favorably to values in the literature. The concept of programming an "arbitrarily large network based on parallelization" is certainly attractive, however I have reservations about whether the platform is as scalable as the authors claim. Accordingly, I cannot recommend publication unless the authors can provide convincing answers to the following questions.

1) The probability of both photons being transmitted through the entire setup is 0.33%. when combined with the intrinsic 1/9 success probability of the scheme is just 0.036%. The authors should comment on this in the main text, and address this issue in the context of scaling this system to multiple gates.

2) The total transmission probability implies an average of 62% transmission per photon per pass (assuming perfect photon pair coupling), if I have my math correct. In reality the transmission per pass is probably higher due to imperfect photon coupling. This is impressive given the number of fiber couplers and EOMs the photons must pass through. However, it is still low compared to passive bulk or integrated beam splitters and phase shifters. Can the authors point to potential future improvements that could increase this transmission? Can the EOMs be made fast enough that the delays can be built in free-space to minimize loss? When raised to the 12th power, the low transmission probability of the element is very detrimental.

3) There is the potential for the short and long paths to experience different loss, which I believe will look like a non-unitary state-dependent loss? While this problem is not unique to the time-bin platform, I think it is exacerbated by the need for free-space fiber coupling. The authors should discuss whether this is an issue, and describe methods to mitigate it. This is probably appropriate to discuss in section III of the supplementary.

Finally, as an option, the authors may want to consider adding a diagram that explains how the Hadamard-(C-NOT) circuit looks in space and time. I.e. an analogous diagram to figure 1. This would be best placed in the supplementary information.

Version 1:

Reviewer comments:

Reviewer #1

(Remarks to the Author)
I thank the authors for taking the suggestions and comments to revise their paper; it became better, but I think it still didn't clearly present their research in the revised manuscript and supplementary material. For reviewers' comments, it seems not to reply clearly. It is difficult to find the revised parts or added parts in the manuscript and the supplementary material from the authors' reply because the authors only reply to reviewers, but do not point out the revised parts in their main text or figures. The replied information didn't appear in the revised paper. I think it is still not good enough to be accepted in the Journal of Nature Communications.

From the section of the SPDC source preparation, we knew that the polarized photon pairs are prepared in free space. Is

there any reason to use the "single-mode fibers", not "polarization-maintaining fibers", for the optical delay? How to stabilize the polarization/phase through the related long (over 1 km *6 round-trip) delay fibers? Does the polarization dispersion through the single-mode fibers also induce the system errors?

Authors compared the same post-selected interferometric scheme in bulk (2003) and integrated optics (2011). These papers are not recent results. Have recent results performed similar experiments?

One of the research degrees of freedom is the time bin. The authors introduced the frequency and orbital angular momentum of light. Are there any time bin-related references?

“Fig. 9 shows the switching sequences applied to EOM 3 by means of the arbitrary signal generator for the four different C-T input states. Overlaid to the time-line, we show the same BS symbols used in Fig. 5 to show the correspondence between voltage and circuital operation. In particular, transmissive operations correspond to applying no voltage, while full reflections correspond to applying a certain voltage V* to the cell. Splittings with reflectivities 1/2 and (+/-)1/3 are implemented applying a positive or negative fraction of V* to the Pockels cell.”
Does it only apply to positive or negative of V* to prepare 1/2, 1/3, -1/3 BS? Should they be set to V*, 1/2V*, 1/3V*, and -1/3V*, respectively?

The TM system used time bins of photons, but the authors used a CW laser to check the EOM3. Is it possible to lose the "time operation information (splitting ratios by time)" for each time bin, not using a pulsed laser?
I think I cannot find the reply to the information in the supplementary material or main text, which was only shown in the revised letter. Can I know the reason? Please correct me if I missed them somewhere.

The authors suggested their photon pairs were not perfectly indistinguishable to calculate the correct and the error probabilities, while suggesting their TM system is perfect operation to the CNOT gate. I think the main purpose of this paper is to demonstrate the TM CNOT gate operation, so it is important to provide the information of the experimental TM system, such as EOM loss, time operation in the BS, SNSPD detection efficiency, dark counts, time-jitter, SPDC coincidence rate…, and show the reader the real performance of the "CNOT" gate. So, there are reference papers that can be used to estimate the bounds of a “logic gate” even though we didn’t perform the full measurements of quantum process tomography.

“Although the degree of indistinguishability is expected to degrade the gate fidelity as it requires two-photon interference in its operation this is not the only limiting facts as in fact the gate fidelity is affected also by higher photon components from the PDC also when the control is initiated in the 0 state and the gate does not rely on partial HOM interference. We also would like to point out that the gate fidelity and the quantum state fidelity used for Bell states are different measures. While the former is obtained by comparing the output coincidence pattern to the expected truth table, the latter relies on the reconstruction of the density operator which is much more sensitive to inaccuracies in the reconstruction. Therefore, the quantum state fidelities may be reduced further than the gate fidelity.”
The authors didn’t reply to the question about the high error in the four generated Bell states. If we consider the error, two results of state fidelities are less than 70%, which is lower than 70.7% to violate the CHSH-Bell inequality. The purity of SPDC photon pairs is not 100% but almost 98%, and the mean photon number is 0.01 “As stated in the main text, pump power is set to a level resulting in a mean photon number of 0.01 for in the signal-idler pair, this ensures that generation events where more than two photons has been generated can be neglected.”.

Please check the first paragraph in the section “SPDC source” in the supplementary material. In the section “Time-multiplexed interferometric scheme” and Figure 2, the authors may have mixed Δτ and Δt, τr and tr. Please check them carefully.

I think Fig.1 in the supplementary material is still not consistent with the main text shown in the section “SPDC source”. There are no Fig. 1 (a) and (b).

In Fig 2 (b), the figure caption is not clear. I think it is the measurement directly after the SPDC photon source.
If possible, the authors can perform the same measurement after the TM system and show the difference in coincidence counts, not normalized coincidences, to compare the difference between the effect of the TM system.

The authors also mentioned this TM system to an arbitrarily large network, so we are also interesting how many photon pairs can be passed after the CNOT gate operation. So, showing the coincidence counts and system loss can help the reader to understand the current system situation.

Reviewer #2

(Remarks to the Author)
I thank the authors for their considered answers to my comments, and apologize for my order of magnitude error when calculating the loss. I found the discussion in the author response to be very useful for me.

However, I don't feel like enough of that discussion has been added to the manuscript. Loss is a big issue for all photonic platforms, not only this paper, as the authors rightly point out. But I don't think this means we can ignore it when writing important papers like this one. I would strongly encourage the authors to add more discussion about loss into the main

paper, and to also introduce opportunities for improving the loss in future versions.

Version 2:

Reviewer comments:

Reviewer #1

(Remarks to the Author)
I want to thank the authors for taking these suggestions and comments into account and revising their manuscripts repeatedly. However, I would also like to note that the reviewer should not be used to check or collect your research and manuscript. So, I really suggest that the authors prepare the perfect manuscript and then submit it in their future research. In fact, judging by this revised draft, the authors still made further revisions to their manuscript, and this is already our second round of peer review for the same paper.

I think the latest revised manuscript has become clearer in presenting its results. But I have other questions and suggestions for the “rebuttal”.
1. Please check the paper title as “Quantum Process Tomography of a Controlled-Phase Gate for Time-Bin Qubits” in PRApplied. Perhaps such studies could serve as references regarding the operation of time-bin qubit logic gates.

2. I assume that the updated analysis was performed on the same experimental dataset, without acquiring additional measurements. While I appreciate that the authors revised their error estimation method and obtained reduced uncertainties, I find it unclear why the reported state fidelities themselves appear to change. In general, for a fixed dataset, modifying the error estimation procedure should only affect the associated uncertainties, but not the reported fidelity values. Do you use a similar method to reconstruct the density matrix and calculate the state fidelity with this reference paper, Phys. Rev. A 64, 052312 (2021)? Maybe I was wrong, I think the state fidelity should not be changed while you do the error calculation, but possible be changed by performing the maximum likelihood estimation. By the way, I don't understand why errors are over 4 times different as shown in the FIG. 3 in the supplementary material? Therefore, I would ask the authors to clarify whether the reconstructed density matrices or the data analysis procedure have been modified in the revised manuscript. I believe this is a very important clarification, demonstrating that the author did indeed conduct the experiment correctly and used appropriate methods to analyze the experimental data in order to present the results. This is because QST is a fundamental experimental measurement in quantum optics.

3. In the Discussion, “Further improvements could be obtained by reducing the fiber length, which would become feasible with faster EOMs.” Maybe I was wrong. If reducing the fiber length, that means the operation speed of the EOM should also be increased. Is it possible to fit your EOM? Is it possible to use the LN modulator to increase the operation speed?

4. While the demonstrated two-qubit operations and Bell-state generation are convincing, several statements in the Discussion regarding scalability, transmission efficiency, and achievable data rates appear to rely on extrapolations that are not quantitatively supported by the presented experimental results. For instance, the projected increase in transmission efficiency to 90% and the claimed factor-of-30 enhancement in data rates are not accompanied by a detailed loss model or system-level analysis. Given that the current setup exhibits an overall efficiency of approximately 21% and relatively low event rates, it is unclear how these projections are derived. Similarly, the claim of a “clear scalability advantage” and the pathway toward “large-scale photonic quantum circuits” would benefit from a more careful discussion of limiting factors such as accumulated loss over multiple round-trips, mode mismatch, and error propagation. I would therefore encourage the authors to either provide quantitative justification for these projections or moderate the strength of these claims to better reflect their speculative nature.

Version 3:

Reviewer comments:

Reviewer #1

(Remarks to the Author)
Thank you for taking my recommendations into account when revising the manuscript. I accepted both the report and the revised manuscript.

Open Access This Peer Review File is licensed under a Creative Commons Attribution 4.0 International License, which permits use, sharing, adaptation, distribution and reproduction in any medium or format, as long as you give appropriate credit to the original author(s) and the source, provide a link to the Creative Commons license, and indicate if changes were made.
In cases where reviewers are anonymous, credit should be given to 'Anonymous Referee' and the source.
The images or other third party material in this Peer Review File are included in the article's Creative Commons license, unless indicated otherwise in a credit line to the material. If material is not included in the article's Creative Commons license and your intended use is not permitted by statutory regulation or exceeds the permitted use, you will need to obtain permission directly from the copyright holder.
To view a copy of this license, visit https://creativecommons.org/licenses/by/4.0/

Response to Reviewers

We appreciate the effort that the reviewers dedicated to providing feedback on our manuscript and are grateful for the insightful comments and improvements to our paper. We have incorporated most of the suggestions of the reviewers and we are now going to provide a point-by-point reply to reviewers.

Reviewers' comments to the Authors:

Reviewer 1:

"I think this is an interesting research topic, namely demonstrating the C-NOT gate using a time-multiplexing (TM) system, even though the same research group has already used this system in quantum walk experiments (only 1/2 beam splitter). The experimental arguments of the two studies were different, so I expected to find a different detailed discussion than in the previous study. However, the manuscript is not clear to follow. I suggest that the author use figures and small subsections to explain 1. how their different beam splitters work with EOM, 2. how to operate the C-NOT gate through the TM system, and 3. how to generate entanglement with the C-NOT gate, and make their structure strongly connected to each part."

Author response:

We thank the reviewer for recognizing the interest of our research. We appreciate the important feedback given about the readability of the manuscript. We address this in the revised manuscript, specifically in the section "Time-multiplexed interferometric scheme" by expanding on the concept underlying the looped architecture and how this gives rise to the time-bin sequence that encodes the interferometer with a particular attention to the operations required to perform both in- and out-coupling and coupling of different time-bins via beam splitter operations. Later in the section we the relate each conceptual operation to the device that carries it out within the experimental setup.

"This is an experimental study, but it didn't clearly show its experimental details, for example, how to generate the SPDC photon source, laser information, how to operate EOMs in each round-trip, the length of the fiber,..., which makes it difficult to judge."

Author response:

We thank the reviewer for the comment, and we agree that the main focus of our study is experimental. Regarding the SPDC source, we have now added to the supplementary material a more detailed description of the photon generation setup and its operation. Regarding the EOM operation, the revised manuscript features a section in the Methods where we describe the EOMs' working principle and operation in terms of driving electronic signals employed. In the revised manuscript the fiber lengths are explicitly stated.

"TM in Fig. 2 is very nice, but it didn't clearly explain how to operate and connect the experimental method with Fig. 1. If the authors can describe each specific part, such as Ref. [21], PRL 104, 050502 (2010), [23], NJP, 18, 063017 (2016), and [26], PRL 111, 150501 (2013), and the related output time-bin as shown in Fig. 1 (b). That would be better for the reader to easily understand how it worked."

Author response:

As already stated in replying to the firs point raised to the reviewer, the experimental setup is now described in larger detail both in the main text and in the methods.

"The operation for each EOM is important, depending on every time-bin photon, so I recommend that authors use additional figures and paragraphs to clearly describe it, or use one specific input to demonstrate how it works. For the time-bin logic gates, they need to use a high-speed optical switch for every time-bin state, but the loss is also limited to their application. What is its operation limit and loss of EOM?"

Author response:

We thank again the reviewer for the comment. As already highlighted in replying to a previous comment from the same reviewer we now include details on the EOM in the methods. Regarding the devices' limitations as we use bulk Pockels cells losses within the EOM are negligible provided that the beam is sufficiently small to fit within the clear aperture of the device. This we ensure using collimators with the appropriate focal length to couple to the fibers used to implement the delays. The real limitation of the EOM is their operation speeds: as we now specifically state in the methods the minimum pulse separation for the in-/out-coupling EOMs and the one in the setup feedback are 50 ns and 150 ns, respectively.

"The authors discussed the errors of input control and target states, 0 and 1. For the TM, authors can check the result for all 6 round-trip distributions for C-NOT gate operation and entanglement generation. Another possible error may arise from the light propagation through long optical fibers and EOMs after 6 round-trip. How are these possible to affect the demonstrated results?"

Author response:

During setup aligment and optimization the performance of each operation performed by EOM 3 has been checked by sending a CW laser in the setup while the EOM was programmed to implement the desired switching sequence. This way we ensure that the correct splitting ratios are implemented for all six roundtrips. As for the effect of propagation in fibers since we ensure that both loops have similar efficiencies we do not expect effects on the interference quality but rather an overall reduction in the transmission probability through the setup. Additionally, since we use photons featuring a spectral width of approximately 1 nm, considering a dispersion of 17ps/(nm*km) at 1550 nm for SMF-28 fibers, and considering that the length of the longest fiber in our setup is about 1.1 km, we expect a temporal broadening of 20 ps per roundtrip. Therefore, after considering that we perform at most 7 roundtrips we expect a total broadening of 140 ps. As this value is at least 2 orders of magnitude smaller than the temporal duration of any EOM operation we perform we do not expect dispersion to significantly impact the setup performance.

"Do the 98% HOM interference visibility and C-NOT (entanglement generation) experiments use the same parameters, mean photon number of ~0.01? The SPDC photon indistinguishability is 98% (HOM), but the gate operation fidelity is 93.8% (Fig. 3) and is limited to 95% by the analysis. What is the main expected difference from the perfect gate? From Fig. 3 and Table I results, it does not seem consistent. For c=0, Fig.3 both are higher than Table I; for c=1, both are lower than Table I. c=1, t=0 case has the max difference. What is the error bar of the operation probabilities of probability in Fig. 3?"

Author response:

The HOM interference scan and the experiment were run with the same mean photon number. In the analysis of the limitation on the achievable fidelity we consider an optimal gate operation, i.e. perfect BS operations carried out by the EOM, and we consider how higher-order photon-number contributions from the PDC state influence the gate operation by giving a valid coincidence pattern in the control-target modes. As these contributions derive from an incorrect input state they will not follow the expected behavior as they do not match the encoding we chose for control and target qubit. However, since our detectors lack photon number resolution we cannot distinguish these spurious contributions. We thank the reviewer for pointing out the missing errors which are now included in the figure's caption.

"The best way to fully reconstruct the gate is quantum process tomography, but it needs lots of measurements. If the author performed these measurements, Phys. Rev. Lett. 94, 160504 (2005)., and Phys. Rev. Lett. 95, 210506 (2005)., it can give the bounds of the logic gate easily."

Author response:

We thank the reviewer for the suggestion, however the measurements presented in the references mentioned by the reviewer have not been performed in our case.

“If authors can provide the data of Fig. 4 (a), it will be easy to confirm the average fidelity of 92.6%.”

Author response:

Upon considering again the pattern shown in the Fig. 4(a) of the original manuscript we realized that showing projections of the four Bell states onto the computational basis does not give a full picture of the quality of the generated states. Therefore this picture in not present in the new version of the manuscript, instead we provide real and imaginary part of all the density operators that were reconstructed in the supplementary material. The main text still features the density operator obtained for the state phi^-.

“From the discussion about the C-NOT gate operation, it showed that the logic gate fidelity is very close to perfect at 95%, and the SPDC photons are also very close to indistinguishability. What is the main reason that the state fidelity of generated Bell states became lower, and the error bar is also large in Fig. 4? If the C-NOT gate works well, is the quality of the specific input state good (input preparation)?”

Author response:

Although the degree of indistinguishability is expected to degrade the gate fidelity as it requires two-photon interference in its operation this is not the only limiting facts as in fact the gate fidelity is affected also by higher photon components from the PDC also when the control is initiated in the 0 state and the gate does not rely on partial HOM interference. We also would like to point out that the gate fidelity and the quantum state fidelity used for Bell states are different measures. While the former is obtained by comparing the output coincidence pattern to the expected truth table, the latter relies on the reconstruction of the density operator which is much more sensitive to inaccuracies in the reconstruction. Therefore, the quantum state fidelities may be reduced further than the gate fidelity.

“The authors talk about the distinguishability and indistinguishability of SPDC photons. I suggest the authors can also discuss more about the C-NOT gate itself to the possible error in the TM system.”

Author response:

We thank the reviewer for the suggestion. We could of course consider imperfections in the operation of the TM setup, for instance the action of EOM 3. However, by postulating an optimal operation and only considering inaccuracies in the input we see that the measured fidelity is close to the maximally expected one thus we can conclude a good performance of the TM setup without making any further assumption.

“I also recommend that authors survey more references about the photonic logic gates in different systems. And also introducing more about the advantages of using the TM system for PQC and other possible applications.”

Author response:

We thank the reviewer for the comment. To the best of our knowledge, we included in the introduction the most relevant implementations exploiting photons. As the main challenge of this implementation is to overcome the non-interacting nature of photons in a TM architecture, introducing references to works that do not employ this strategy would be out of the scope of our study. Regarding the use of TM its main advantage is the inherent scalability which is already highlighted in the references cited in this work.

" If possible, please reconsider the structure of the manuscript; the most recently submitted version is difficult to follow. For example, divide the results section into several subsections and ensure a natural transition between them, including appropriate figures and captions."

Author response:

We thank the reviewer for the comment. Although we kept the main subdivision of the old manuscript in the new one, we are confident that the additional information provided will improve its readability.

"The material in the supplemental information is strongly related to the main text. I think it is better to move it to the method section or the main text."

Author response:

We thank the reviewer for the comment. In the new manuscript some if the information has been moved and/or added to the methods.

Reviewer 2:

"The probability of both photons being transmitted through the entire setup is 0.33%. when combined with the intrinsic 1/9 success probability of the scheme is just 0.036%. The authors should comment on this in the main text, and address this issue in the context of scaling this system to multiple gates."

Author response:

We thank the reviewer for the comment, however, we would like to point out a few issues with the estimate for the scheme success probability given by the reviewer. As stated in the manuscript the efficiency of the TM setup for a single pass is 81%. Considering the six roundtrips this means that after input state generation and C-Not a single photon reaches the detection stage with probability of 28%. Given a detection efficiency of the SNPDS and imperfections in the couplings to the detection fibers we can consider an efficiency for the detection stage of 75%. This means that the total efficiency for a single photon amounts to 21%, which in turn means a two-photon probability of 4%. Then considering that of all two photon events only 1/9th correspond to successful instances of the gate the scheme probability is 0.4%, hence at least a factor of 10 larger than the one estimated by reviewer 2. If we do not consider the detection efficiency, which is not specific to the scheme, we find that the TM scheme with the current loop efficiencies have a success rate of ~0.9%.

Aside from this, the point raised by the reviewer is a crucial one and it is not only pertinent to our implementation, but rather to any photonic system. In fact, even an ideal setup would be limited to the inherent 1/9 success rate by the C-NOT design, which already poses a serious limit to size of the circuit that can be realized. Nonetheless, the success rate of the single gate may be enhanced adopting multiplexing and feed-forward, i.e.: using many copies of the probabilistic gate to enhance the success rate and being able of collecting the output of the successful gate and forward it to the next computational stage. In order to implement gate copies running in parallel, an optical circuit must be equipped with a large number of inputs and outputs. As highlighted in the revised manuscript, our TM interferometer has the potentiality of realizing a large network simply by tailoring the lengths of the fibers maintaining the amount of active elements constant. This is not the case in other prominent platforms within photonics, as for instance in path encoded systems, where the circuit dimension is dictated by the physical size of the photonic chip, while the system operation requires a number of components (directional couplers and phase shifters) growing with the number of inputs, which increases the chance for imperfections and crosstalk. Additionally, for this kind of systems, the circuit must be entirely programmed prior to the actual evolution and a partial readout of a subset of mode is not possible. Instead, our in- and out-coupling EOMs can be programmed to act only a subset of time-bins which can be analysed by a fast external logic which could then determine the next operations for all EOMs, rendering possible to include feed-forward in the system. For this set of reasons, we are convinced that our system would be the most suited one to overcome the probabilistic nature of photonic two qubit gates and enable the realization of large quantum circuits.

“The total transmission probability implies an average of 62% transmission per photon per pass (assuming perfect photon pair coupling), if I have my math correct. In reality the transmission per pass is probably higher due to imperfect photon coupling. This is impressive given the number of fiber couplers and EOMs the photons must pass through. However, it is still low compared to passive bulk or integrated beam splitters and phase shifters. Can the authors point to potential future improvements that could increase this transmission? Can the EOMs be made fast enough that the delays can be built in free-space to minimize loss? When raised to the 12th power, the low transmission probability of the element is very detrimental.”

Author response:

As the reviewer correctly points out transmission efficiency within the TM setup is a critical parameter. The setup used for this work features 81% loop efficiency, however a newer iteration of the setup with an improved mode matching shows loop efficiencies of 85%. This value would be improved by adopting shorter fibers. This would be rendered possible by EOMs with higher speeds. For instance, the EOM that carries out the BS operations in the setup has a bandwidth limited to 6.7 MHz, hence the time-bin separation of 170 ns. However, within a few years analogous devices with speeds up to 100MHz might be available. This would reduce the timing requirement by a factor of 10, which would come with the twofold advantage of increasing transmission efficiencies up to 90% and increase the experimental data rate.

“There is the potential for the short and long paths to experience different loss, which I believe will look like a non-unitary state-dependent loss? While this problem is not unique to the time-bin platform, I think it is exacerbated by the need for free-space fiber coupling. The authors should discuss whether this is an issue, and describe methods to mitigate it. This is probably appropriate to discuss in section III of the supplementary.”

Author response:

As the reviewer correctly points out, there is a potential for imbalances in the loop efficiencies. However, as also stated in the new manuscript, the setup alignment is carried out in a way that ensures that all loop efficiencies are comparable. The fact that we obtain an high fidelity for the gate is also an indicator of the fact that the setup alignment is optimal.

“Finally, as an option, the authors may want to consider adding a diagram that explains how the Hadamard-(C-NOT) circuit looks in space and time. I.e. an analogous diagram to figure 1. This would be best placed in the supplementary information.”

Author response:

We thank the reviewer for the suggestion. As the circuit operation in time is an important aspect of our work we added the diagram both for the plain C-NOT and for the Bell state generation circuit in the main text.

Response to Reviewers

We sincerely appreciate the time and effort the reviewers have devoted to evaluating our work. We are grateful for the insightful comments, which have helped us identify and address a few inconsistencies between the main text and the supplementary information, and have enabled us to improve the clarity and overall quality of our work

Below, we provide a detailed point-by-point response to the reviewers' comments.

Replies to the Reviewers' comments to the Authors:

Reviewer 1

Comment 1:

"I thank the authors for taking the suggestions and comments to revise their paper; it became better, but I think it still didn't clearly present their research in the revised manuscript and supplementary material. For reviewers' comments, it seems not to reply clearly. It is difficult to find the revised parts or added parts in the manuscript and the supplementary material from the authors' reply because the authors only reply to reviewers, but do not point out the revised parts in their main text or figures. The replied information didn't appear in the revised paper. I think it is still not good enough to be accepted in the Journal of Nature Communications"

Authors' response:

We thank the reviewer for acknowledging the improvements in the revised manuscript. We apologize for not having indicated the changes and additions with sufficient clarity.

To avoid further confusion and to facilitate the review process, we are providing, in addition to this point-by-point response and the revised manuscript, versions of the files in which all modifications relative to the previous submission are clearly highlighted.

Comment 2:

"From the section of the SPDC source preparation, we knew that the polarized photon pairs are prepared in free space. Is there any reason to use the "single-mode fibers", not

"polarization-maintaining fibers", for the optical delay? How to stabilize the polarization/phase through the related long (over 1 km *6 round-trip) delay fibers? Does the polarization dispersion through the single-mode fibers also induce the system errors?"

Authors' response:

In principle, polarization-maintaining (PM) fibers could indeed be used to implement the delays, thereby eliminating the need for the pre-compensation waveplates currently employed to preserve the input polarization. However, PM single-mode fibers exhibit attenuations of up to 0.5 dB/km at 1550 nm. Given the length of our delay lines—and even neglecting additional losses due to imperfect alignment or mode mismatch—this would restrict the loop efficiency to approximately 89%. By contrast, standard single-mode fibers of the same length, with an attenuation of 0.17 dB/km, limit the efficiency to about 96%. Since the loop efficiency affects the overall efficiency exponentially after n round trips, the use of non-PM single-mode fibers is clearly advantageous.

Although the reviewer correctly notes that the delay fibers may introduce phase instabilities, active stabilization against these effects is not required in our case. This is due to two considerations. First, a single experimental run lasts no more than 30 µs, which is significantly shorter than the typical timescale of temperature fluctuations. We can therefore safely assume that the phase accumulated in the fibers remains constant during each individual run. Second, interference at a given time bin occurs only when the light has traversed both fibers the same number of times. As a result, prior to recombination in the feedback loop, both polarization components accumulate identical phase shifts, rendering the interference insensitive to fiber-induced phase fluctuations.

A similar argument applies to dispersion. Because interference requires that the light propagate through the loops the same number of times, both polarizations experience identical temporal broadening. Consequently, neither the interference visibility nor the indistinguishability of the particles is degraded by dispersive effects in the fibers.

Comment 3:

"Authors compared the same post-selected interferometric scheme in bulk (2003) and integrated optics (2011). These papers are not recent results. Have recent results performed similar experiments?"

Authors' response:

We thank the reviewer for raising this point. To the best of our knowledge, in bulk optics no significant new results have been reported since the original implementation by O'Brien *et al.* (2003).

Upon re-examining the literature on integrated photonics, we identified more recent efforts aimed at reducing the footprint of integrated circuits implementing two-qubit gates [Phys. Rev. Lett. 126, 130501 (2021); Sci. Adv. 10.1126/sciadv.adg6685 (2023)], achieving gate fidelities comparable to those reported in our work.

Since we believe it is important to emphasize that the development of photon-based two-qubit gates remains an active area of research, we have included these references

in the revised manuscript, both in the Introduction and in Section IC (“Demonstration of the TM C-NOT gate”).

Comment 4:

“One of the research degrees of freedom is the time bin. The authors introduced the frequency and orbital angular momentum of light. Are there any time bin-related references?”

Authors’ response:

We thank the reviewer for raising this point. To the best of our knowledge, the only other demonstration to date of a temporally encoded two-qubit gate is that reported by Humphreys *et al.* (2014), which we cite in the introduction of our manuscript:

“A possible TM scheme capable of implementing single-and two-qubit gates has already been demonstrated by Humphreys et al. in [26], where an interferometer featuring only static linear optical elements has been used to realise a post-selected C-PHASE gate.”

The C-PHASE gate demonstrated in the referenced work achieved a fidelity of 84%. Although this value is lower than that of our gate, the two results are not directly comparable, as the respective operations rely on different optical circuits.

Comment 5:

“ “Fig. 9 shows the switching sequences applied to EOM 3 by means of the arbitrary signal generator for the four different C-T input states. Overlaid to the time-line, we show the same BS symbols used in Fig. 5 to show the correspondence between voltage and circuital operation. In particular, transmissive operations correspond to applying no voltage, while full reflections correspond to applying a certain voltage V* to the cell. Splittings with reflectivities 1/2 and (+/-)1/3 are implemented applying a positive or negative fraction of V* to the Pockels cell.”
Does it only apply to positive or negative of V* to prepare 1/2, 1/3, -1/3 BS? Should they be set to V*, 1/2V*, 1/3V*, and -1/3V*, respectively?”

Authors’ response:

The reviewer is correct in raising this technical point. The relationship between the splitting ratio and the voltage applied to the Pockels cell is not strictly linear. Specifically, for an applied voltage V, the EOM implements a reflection

$$R = \sin^2\left(\frac{\pi}{2V^*}\,V\right).$$

Inverting this relation, $R = 1$ and $R = \frac{1}{2}$ correspond to $V = V^*$ and $V = \frac{1}{2}V^*$, respectively, while $R = \pm 1/3$ corresponds to $V = \pm 0.39V^*$.

We agree that providing this information improves clarity regarding the operation of the setup. Accordingly, we have included both the functional dependence given above and the corresponding voltage fractions in Subsection A2 of the Methods, where the operation of EOM 3 is described.

Comment 6:

“The TM system used time bins of photons, but the authors used a CW laser to check the EOM3. Is it possible to lose the "time operation information (splitting ratios by time)" for each time bin, not using a pulsed laser?
I think I cannot find the reply to the information in the supplementary material or main text, which was only shown in the revised letter. Can I know the reason? Please correct me if I missed them somewhere.”

Authors’ response:

We thank the reviewer for pointing this out and acknowledge that we omitted some key information necessary to understand how the EOM operation is verified.

We do not lose temporal information about the splittings because, to characterize them, we record a histogram of the system output triggered by the same signal used to initiate the experiment. When a horizontally polarized CW laser is injected into the setup, the resulting histogram exhibits a series of dips corresponding to the activations of EOM 3. This allows us to verify and optimize each splitting ratio by evaluating the contrast of each dip, defined as the ratio between the number of counts inside and outside the dip.

Temporal synchronization is achieved by applying a global delay to the operation sequence so that the first switching event matches the corresponding time bin. We also ensure that the relative timing between successive dips agrees with the expected values determined by τ_r and $\Delta\tau$.

In the revised version of our manuscript we now provide this information in Subsection A2 of the Methods.

Comment 7:

“The authors suggested their photon pairs were not perfectly indistinguishable to calculate the correct and the error probabilities, while suggesting their TM system is perfect operation to the CNOT gate. I think the main purpose of this paper is to demonstrate the TM CNOT gate operation, so it is important to provide the information of the experimental TM system, such as EOM loss, time operation in the BS, SNSPD detection efficiency, dark counts, time-jitter, SPDC coincidence rate..., and show the reader the real performance of the "CNOT" gate. So, there are reference papers that can be used to estimate the bounds of a “logic gate” even though we didn’t perform the full measurements of quantum process tomography.”

Authors’ response:

The reviewer is correct in noting that a central objective of the paper is to demonstrate the operation of the TM gate. Equally important, however, is that our implementation constitutes, to the best of our knowledge, the first realization of time multiplexing in a platform capable of scaling to many modes without the resource overhead typical of path-encoded approaches, while remaining rapidly reconfigurable and therefore compatible with feed-forward operations.

We know from the HOM interference visibility of 98% that the photons exhibit a distinguishability of no more than 2%. In addition, the input state is not ideal in photon number, since we employ signal–idler pairs without heralding. As a result, there is a finite probability of generating more than two photons, leading to spurious control–target events that can be registered as gate errors.

Taking these intrinsic and unavoidable features of the input resource into account, even an ideal gate would yield an operation fidelity in the computational basis bounded by 95%. We emphasize that this bound is not specific to our circuit, but would apply to any experimental implementation relying on the same type of input resource.

Because the achieved fidelity is 93.8%, it is obvious that the circuit is not perfect. However, comparing the measured 93.8% against the expected 95%, one finds that our circuit's operation fidelity is around 98.7%. To avoid possible misunderstandings, we have rephrased the relevant passage in Section IC ("Demonstration of the TM-CNOT gate").

We thank the reviewer for pointing out that the manuscript would benefit from a more detailed description of the experimental parameters. In Section IB ("Experimental Setup"), we now explicitly report the transmission of the EOMs (≥ 99%), the pair-generation probability (1%), the waveguide length (2.5 cm) and propagation losses (1.2 dB/cm), the detection efficiency (> 90%), the detector timing jitter (~70 ps), the impact of dark counts, and the overall system efficiency from generation to detection (21%). Together with the parameters already included in the manuscript, we believe this provides a comprehensive and transparent overview of the experimental setup.

Regarding the reviewer's comment on the "time operation in the BS," as discussed in our response concerning the temporal operation of EOM3 (which can be verified using a CW laser), the beam-splitter (BS) operation is optimized by analyzing a time-resolved histogram. This allows us to ensure that the EOM acts on the intended time bins and implements the desired splitting ratios.

Comment 8:

" "Although the degree of indistinguishability is expected to degrade the gate fidelity as it requires two-photon interference in its operation this is not the only limiting facts as in fact the gate fidelity is affected also by higher photon components from the PDC also when the control is initiated in the 0 state and the gate does not rely on partial HOM interference. We also would like to point out that the gate fidelity and the quantum state fidelity used for Bell states are different measures. While the former is obtained by comparing the output coincidence pattern to the expected truth table, the latter relies on the reconstruction of the density operator which is much more sensitive to inaccuracies in the reconstruction. Therefore, the quantum state fidelities may be reduced further than the gate fidelity."
The authors didn't reply to the question about the high error in the four generated Bell states. If we consider the error, two results of state fidelities are less than 70%, which is lower than 70.7% to violate the CHSH-Bell inequality. The purity of SPDC photon pairs is not 100% but almost 98%, and the mean photon number is 0.01 "As stated in the main text, pump power is set to a level resulting in a mean photon number of 0.01 for in the signal-idler pair, this ensures that generation events where more than two photons has been generated can be neglected."."

Authors' response:

We thank the reviewer for emphasizing this point. Upon re-examining the method used to determine the uncertainties, we found that our analysis of error was indeed too conservative and it led to an overestimation of the errors associated with the fidelities.

Given the experimentally reconstructed and target density operators, ρ_e and ρ_t, the quantum state fidelity between the two was calculated using the general expression

$$F = \left(\mathrm{Tr}\sqrt{\sqrt{\rho_t}\,\rho_e\,\sqrt{\rho_t}}\right)^2 .$$

In this form, it is not straightforward to derive a closed analytical dependence of F on the parameters of ρ_e. We therefore estimated the uncertainty using a Monte Carlo approach. Starting from ρ_e and its associated errors, we generated an ensemble of density operators whose elements were sampled from normal distributions with mean values and standard deviations equal to the corresponding elements and uncertainties of ρ_e.

The uncertainty on F was then evaluated as the standard deviation of the fidelities computed for the generated density operators. This procedure yielded the error values reported in the first and second versions of the manuscript.

However, in our case the Monte Carlo approach can be avoided because the target density operators are pure and therefore have the form $\rho_t = \mid \psi\rangle\langle\psi \mid$. Substituting this expression into the definition of the fidelity yields the simplified relation

$$F = \langle\psi \mid \rho_e \mid \psi\rangle.$$

In our tomography setup, we measure the correlations

$$s_{\mathrm{i,j}} = \langle\sigma_i \otimes \sigma_j\rangle,$$

with $i = 0, x, y, z$ and σ_0 denoting the 4×4 identity. The experimentally reconstructed density operator can be written as

$$\rho_e = \sum_{i,j} \frac{s_{i,j}}{4}\, \sigma_i \otimes \sigma_j.$$

Accordingly, the fidelity becomes

$$F = \sum_{i,j} \frac{s_{i,j}}{4} \langle\psi \mid \sigma_i \otimes \sigma_j \mid \psi\rangle.$$

Given the uncertainties $\Delta s_{i,j}$ associated with the measured correlations, the uncertainty in F can be obtained via standard Gaussian error propagation,

$$\Delta F = \sqrt{\sum_{i,j} \frac{\langle\psi \mid \sigma_i \otimes \sigma_j \mid \psi\rangle^2}{16}\, \Delta s_{\mathrm{i,j}}^2}.$$

Using this analytical expression, we recalculated the fidelities and their associated uncertainties for each state. The updated values (also reported in the main text and

supplementary Information) are:
$F_{\psi^+} = (85.6 \pm 3.0)\%$,
$F_{\psi^-} = (78.1 \pm 2.8)\%$,
$F_{\phi^+} = (80.3 \pm 3.0)\%$,
$F_{\phi^-} = (85.0 \pm 2.4)\%$.

With these updated numbers, the four Bell states' fidelities remain consistently above the non-classicality bound.

Comment 9:

"Please check the first paragraph in the section "SPDC source" in the supplementary material. In the section "Time-multiplexed interferometric scheme" and Figure 2, the authors may have mixed Δτ and Δt, τr and tr. Please check them carefully."

Authors' response:

We thank the reviewer for spotting these inconsistencies which are now fixed in the revised manuscript.

Comment 10:

"I think Fig.1 in the supplementary material is still not consistent with the main text shown in the section "SPDC source". There are no Fig. 1 (a) and (b)."

Authors' response:

We thank the reviewer for pointing out this inconsistency in the figure references. The issue arose because, in an intermediate version of the manuscript, what is now presented as Fig. 1 consisted of two separate subfigures. This has been corrected in the revised Supplementary Information (Section "SPDC Source").

Comment 11:

"In Fig 2 (b), the figure caption is not clear. I think it is the measurement directly after the SPDC photon source.
If possible, the authors can perform the same measurement after the TM system and show the difference in coincidence counts, not normalized coincidences, to compare the difference between the effect of the TM system."

Authors' response:

The reviewer is correct in noting that the photon indistinguishability is measured directly after the source.

In principle, one could devise an experiment in which the same HOM interference scan is performed after the TM setup. This would require injecting photons from the source into the setup and implementing two round trips, while programming EOM 3 to flip the polarization of each photon upon arrival at the feedback. In this configuration, the H (V) photon would first traverse the short (long) delay and then the long (short) delay, thereby re-synchronizing the photon pair. At that point, an analogous polarization-based HOM measurement could be carried out. However, the degree of indistinguishability would not be altered by the TM setup. For the photons to be synchronized, each must traverse the long and short loops the same number of times. Consequently, as discussed in our

response to the reviewer's first comment, both photons accumulate identical phase shifts and experience the same temporal delay, dispersion, and losses.

If instead the reviewer is suggesting a configuration in which HOM interference occurs before the TM setup but the photons are detected afterward, then no effect of the setup—apart from an overall reduction in two-photon events due to losses—would be observable. In such a scenario, the interference visibility would be determined solely by the photons' indistinguishability prior to entering the TM setup.

As a final remark, although it might be interesting to experimentally verify this by performing the proposed measurements, this is not feasible in the immediate future. The laboratory has recently been relocated to a new building and, although the TM setup has been rebuilt, no SPDC source is currently available. Moreover, the rebuilt setup incorporates updated components, making it impossible to reproduce exactly the same experimental conditions.

In order to clarify how the polarization HOM experiment is performed, we provide an updated version of Fig. 1 in the supplementary information.

Comment 12:

"The authors also mentioned this TM system to an arbitrarily large network, so we are also interesting how many photon pairs can be passed after the CNOT gate operation. So, showing the coincidence counts and system loss can help the reader to understand the current system situation."

Authors' response:

We thank the reviewer for raising this important point. The experiment operates at a repetition rate of 21 kHz, with a pair-generation probability of 1%. The total transmission including losses in the source detection efficiency and a single pass in the TM setup is 21%. Additionally in order to complete the six round trips, both photons experience the loop efficiency five times. After the full evolution, we record approximately 4,000 coincidences per hour, which are reduced to about 450 per hour after post-selection.

For a similar experiment involving three photons under the same experimental conditions, the total count rate would decrease by approximately an order of magnitude, making the measurement significantly more demanding. In this context, the loop efficiency plays a crucial role, as it reduces the overall system efficiency exponentially with the number of round trips.

A straightforward strategy to improve this parameter would be to adopt shorter fibers, which in turn would require faster EOMs. For example, EOM 3 is currently bandwidth-limited to 6.7 MHz; however, devices with speeds up to 100 MHz are expected to become available in the coming years. This would allow both the time-bin separation and the round-trip duration to be reduced by roughly an order of magnitude, enabling operation at repetition rates up to ten times higher.

We are confident that the corresponding reduction in fiber lengths, combined with improved alignment and mode matching, could increase transmission efficiencies to approximately 90%. Together with the higher repetition rate, this would enhance the two-photon coincidence rate by roughly a factor of 30, making the same experiment feasible within minutes.

Therefore, although the current implementation has practical limitations, foreseeable technical advances provide a clear pathway toward scaling the approach to larger photon numbers and more complex circuits.

Following the reviewer's suggestion, we now explicitly report the number of two-photon events observed after the CNOT gate in Subsection IC ("Demonstration of the TM CNOT Gate"). We also recognize the importance of discussing potential improvements to the TM architecture in order to clearly present both the current performance of the system and its future prospects. Accordingly, the considerations outlined here have been incorporated into Section II ("Discussion") of the revised manuscript.

Reviewer 2

Comment 1:

"I thank the authors for their considered answers to my comments, and apologize for my order of magnitude error when calculating the loss. I found the discussion in the author response to be very useful for me."

Authors' response:

We thank the reviewer for their thoughtful follow-up and for the positive feedback on our responses. We greatly appreciate the constructive and collegial exchange, and we are pleased that our clarifications were helpful.

Comment 2:

"However, I don't feel like enough of that discussion has been added to the manuscript. Loss is a big issue for all photonic platforms, not only this paper, as the authors rightly point out. But I don't think this means we can ignore it when writing important papers like this one. I would strongly encourage the authors to add more discussion about loss into the main paper, and to also introduce opportunities for improving the loss in future versions."

Authors' response:

We thank the reviewer for recognizing the importance of our work. Following their suggestion, we have incorporated the key points of the discussion on losses and system capabilities that were provided in our previous response into Section II (Discussion) of the main manuscript.

Response to the Reviewer's comments

We sincerely appreciate the time and effort the reviewers have devoted to evaluating our work. We are grateful for the comments by Reviewer 1 which have been useful to clarify some remaining points and improve the quality of the manuscript

Below, we provide a detailed point-by-point response to the points raised by Reviewer 1.

Comment 1:

"Please check the paper title as "Quantum Process Tomography of a Controlled-Phase Gate for Time-Bin Qubits" in PRApplied. Perhaps such studies could serve as references regarding the operation of time-bin qubit logic gates."

Authors' reply:

We thank the reviewer for bringing this reference to our attention, as it provides valuable insight into the implementation of logic gates for time-bin qubits. While it is not possible for us to apply the same methods used in that work to perform a quantum process tomography—since this would require additional measurements that, as mentioned in one of our previous responses, cannot be carried out in the near future due to logistical constraints—the reference nonetheless illustrates that time has already been successfully used as a degree of freedom to implement quantum gates and gives us the opportunity of highlighting the main differences between the scheme presented in this work and in ours, namely the fact that our scheme requires only a single spatial mode to multiplex several time-bins which can, in principle, encode many qubits. For this reason, we decided to cite it in the introduction of the revised manuscript.

Comment 2:

"I assume that the updated analysis was performed on the same experimental dataset, without acquiring additional measurements. While I appreciate that the authors revised their error estimation method and obtained reduced uncertainties, I find it unclear why the reported state fidelities themselves appear to change. In general, for a fixed dataset, modifying the error estimation procedure should only affect the associated uncertainties, but not the reported fidelity values. Do you use a similar method to reconstruct the density matrix and calculate the state fidelity with this reference paper, Phys. Rev. A 64, 052312 (2021)? Maybe I was wrong, I think the state fidelity should not be changed while you do the error calculation, but possible be changed by performing

the maximum likelihood estimation. By the way, I don't understand why errors are over 4 times different as shown in the FIG. 3 in the supplementary material? Therefore, I would ask the authors to clarify whether the reconstructed density matrices or the data analysis procedure have been modified in the revised manuscript. I believe this is a very important clarification, demonstrating that the author did indeed conduct the experiment correctly and used appropriate methods to analyze the experimental data in order to present the results. This is because QST is a fundamental experimental measurement in quantum optics."

Authors' reply:

We are pleased that the reviewer appreciates the revision of our error estimation, and they are correct in assuming that the updated analysis has been performed on the same dataset used previously. As reported in the corresponding section of the Methods ("Two-Qubit Tomography Setup"), the density operators are reconstructed from coincidence measurements performed in different bases, which are used to estimate the correlations of tensor products of Pauli operators. These quantities correspond, up to a normalization factor, to the Stokes parameters discussed in Phys. Rev. A 64, 052312 (2021).

As noted in our previous reply, the fidelities originally reported were calculated using the formula

$F = (\mathrm{Tr}\sqrt{\sqrt{\rho_t}\,\rho_e\,\sqrt{\rho_t}})^2,$

whereas in the updated analysis we recomputed them using

$F = \langle\psi \mid \rho_e \mid \psi\rangle.$

From a theoretical standpoint, these expressions are equivalent when substituting $\rho_t = \mid \psi\rangle\langle\psi \mid$, and therefore they should yield identical fidelity values when applied to ρ_e. In practice, however, we observe slight differences.

From one side, since the new uncertainties feature a different number of significant digits the fidelities had to be rounded accordingly which can already lead to minor changes in the values with respect to the old ones.

In addition, the numerical routine used to evaluate the matrix square root, we used the SciPy method fractional_matrix_power, relies on Schur decomposition which can lead to numerical errors in case the eigenvalues of the matrix of the matrix involved are close or equal to zero.

This is especially relevant in our case since the target density operators ρ_t represent pure states, and therefore have always three of the four eigenvalues that exactly equal zero.

By contrast, the updated method only requires matrix–vector multiplications and scalar summations, which are numerically more stable and less prone to computational errors than evaluating fractional matrix powers.

Regarding the reduction of the error bars, as mentioned in our previous reply, the earlier estimation relied on a Monte Carlo method. Such approaches can overestimate uncertainties due to finite sample size effects and potentially inhomogeneous sampling of the space of possible density operators.

In addition to this, the instabilities due to the calculation of the square root of the density operators cause a larger variability in the generated sample of fidelities and therefore enhance the variance associated to the sample.

By contrast, the updated error estimation follows the same procedure discussed in Phys. Rev. A 64, 052312 (2021) for quantities such as the Von Neumann entropy, Linear entropy and other functions of the reconstructed density operator. Although the quantities explicitly discussed in the referenced work do not include the fidelity, since it is possible to express it as a function of the Stokes parameters of the density operator the errors can be obtained as:

$$\Delta F^2 = \sum_{i,j} \left(\frac{\partial F}{\partial s_{i,j}}\right)^2 \Delta s_{i,j}^2$$

Which leads to the expression of eq. 7 shown in our Supplementary material, where the $\Delta s_{i,j}$ are the errors derived propagation the Poissonian error associated to the coincidences.

Comment 3:

“In the Discussion, “Further improvements could be obtained by reducing the fiber length, which would become feasible with faster EOMs.” Maybe I was wrong. If reducing the fiber length, that means the operation speed of the EOM should also be increased. Is it possible to fit your EOM? Is it possible to use the LN modulator to increase the operation speed?”

Authors’ reply:

We thank the reviewer for raising this important point concerning the trade-offs and technical challenges in scaling the TM system. The key parameter is the time-bin separation, Δτ, set by the length difference between the two fibers.

EOM 3 has a bandwidth of 6.7 MHz, corresponding to a minimum pulse separation of about 150 ns, which is sufficient to independently address all time bins in the current setup. However, this modulator would not be suitable if both delay fibers were shortened by the same factor, since the time-bin separation would also decrease.

A modified configuration with shorter delay lines but unchanged temporal separation could nevertheless be implemented. For example, fiber lengths of 525 m and 560 m would preserve Δτ while reducing the round-trip time by a factor of two. Accounting for fiber attenuation, the transmission in the long fiber would increase from about 95% to approximately 97.5%. While this is a 2.5% improvement per round trip, over the six passes required in the experiment it would lead to an overall transmission increase of about 16%. In addition, the repetition rate of the experiment could be doubled.

This improvement would come at the cost of halving the number of supported modes. In our case this would still be acceptable, as the system would support about 30 modes, whereas only four are required to implement the CNOT gate.

Maintaining or increasing the number of modes would require reducing the time-bin separation, which in turn would necessitate faster EOMs. As noted by the reviewer, integrated modulators based on lithium niobate (LN) or thin-film lithium niobate (TFLN) are promising candidates, as they can reach bandwidths in the tens or hundreds of GHz.

However, integrating such devices into our architecture is challenging due to their typical fiber-to-fiber insertion losses (about 2.5–4 dB) and their polarization-dependent electro-optic efficiencies, which are incompatible with the polarization encoding used in our setup.

Comment 4:

“While the demonstrated two-qubit operations and Bell-state generation are convincing, several statements in the Discussion regarding scalability, transmission efficiency, and achievable data rates appear to rely on extrapolations that are not quantitatively supported by the presented experimental results. For instance, the projected increase in transmission efficiency to 90% and the claimed factor-of-30 enhancement in data rates are not accompanied by a detailed loss model or system-level analysis. Given that the current setup exhibits an overall efficiency of approximately 21% and relatively low event rates, it is unclear how these projections are derived. Similarly, the claim of a “clear scalability advantage” and the pathway toward “large-scale photonic quantum circuits” would benefit from a more careful discussion of limiting factors such as accumulated loss over multiple round-trips, mode mismatch, and error propagation. I would therefore encourage the authors to either provide quantitative justification for these projections or moderate the strength of these claims to better reflect their speculative nature.”

Authors’ reply:

We thank the reviewer for recognizing the validity of the demonstration of the two-qubit operation and the Bell-state generation. We agree that a more quantitative discussion of the factors limiting the present setup further strengthens the manuscript. Accordingly, in addition to correcting several minor inconsistencies in the terminology used in the discussion in the main text, we have added a dedicated section to the Supplementary Material that analyzes the impact of losses. In this section, we relate the experimental parameters to the achievable two-photon rates and outline how improved mode matching and reduced fiber lengths are expected to significantly enhance the data rates in future implementations of the setup.
